# Factors Associated with Unmet Dental Care Needs among Korean Adult Cancer Survivors: Cross-Sectional Analysis of the 2016–2018 KNHANES

**DOI:** 10.3390/healthcare10081563

**Published:** 2022-08-18

**Authors:** So-Yeong Kim, Sun-A Lim

**Affiliations:** 1 Department of Preventive Medicine, Chosun University Medical School, Gwangju 61452, Korea; 2 Department of Dental Hygiene, Songwon University, Gwangju 61756, Korea

**Keywords:** adult, cancer survivor, dental care, dental health surveys, oral health, Korea

## Abstract

This study aimed to identify the factors associated with unmet dental care needs among Korean adult cancer survivors. This cross-sectional study used data from the seventh (2016–2018) Korea National Health and Examination Survey. It included 339 adult cancer survivors. Participants’ experience of unmet dental care needs was assessed using a health questionnaire survey. Moreover, the subjective oral health status (i.e., toothache) and behavior (i.e., toothbrushing and oral examination) were assessed through oral health interviews. The data were analyzed using descriptive statistics, chi-squared test, and logistic regression analysis. The rate of experience of unmet dental care needs among cancer survivors was 29.5%. Cancer survivors with limitations in performing daily activities of living were more likely to experience unmet dental care needs than cancer survivors without activity limitations ([aOR] = 2.14, [95%CI] = 1.04–4.40). Moreover, cancer survivors who did undergo oral examination within the past year were more likely to experience unmet dental care needs than cancer survivors who underwent oral examination ([aOR] = 2.49, [95%CI] = 1.22–5.07). Korean cancer survivors experienced unmet dental care needs when they did not receive an oral examination or had activity limitations. This study’s findings provide insight into social and behavioral factors associated with unmet dental care needs among Korean cancer survivors.

## 1. Introduction

Cancer is an important global public health issue. Cancer patients require extensive treatment and may show poor health outcomes [1]. The number of cancer survivors has been increasing owing to improvements in cancer treatment methods with advances in medical technology [2]. However, cancer survivors face the risk of oral and dental diseases, which may manifest based on the patient’s general condition [3].

The oral complications of cancer treatment include acute complications, such as mucositis, infection, reduced salivary secretion, altered taste, and pain, as well as chronic complications, such as soft and hard tissue necrosis, dental caries, and disruption in dental growth and development [3]. Oral complications caused by cancer can, therefore, significantly affect the oral health, general health, and quality of life of patients, while deterioration of oral health before, during, and after cancer treatment can impact the treatment outcomes of cancer survivors [4]. Such cancer treatment-related complications emphasize the need for oral care after cancer treatment to guarantee optimal oral and general health.

Oral care protocols for cancer survivors typically include oral evaluation and general oral care education, including dental treatments, toothbrushing methods, flossing, and other oral care techniques [5]. Furthermore, since oral complications are linked to cancer survivors’ quality of life, reducing these complications is vital.

Cancer survivors have been assessed for various unmet needs and sequelae of long-term treatment, including fear of cancer relapse, concerns about the well-being of family members, and the desire to return to work [6,7,8]. However, oral complications caused by cancer and cancer treatment remain under-reported, under-recognized, and under-treated [9]. There have been many studies on the unmet psychosocial, emotional, physical, and economic needs of cancer survivors [10,11,12]. According to these previous studies, oral complications of cancer survivors increase, and quality of life decreases. However, there is a high probability of experiencing unmet dental care needs due to the fear of visiting the dentist. However, studies on their unmet dental care needs are scarce.

Accordingly, this study aimed to identify the factors associated with the unmet dental care needs among cancer survivors.

## 2. Materials and Methods

This study is reported in accordance with the Strengthening the Reporting of Observational Studies in Epidemiology guidelines (STROBE) [13].

### 2.1. Research Data

This study used data from the seventh (2016–2018) Korea National Health and Examination Survey (KNHANES) conducted by the Korea Disease Control and Prevention Agency, formerly known as the Korea Centers for Disease Control and Prevention (KCDC). KNHANES is a cross-sectional survey designed to collect nationally representative and reliable data regarding the health status, the prevalence of chronic diseases, and food and nutrient intake status of Korean citizens. The stratified cluster sampling method was used to select the nationally representative samples. In KNHANES, data were collected by performing a health questionnaire survey and health examinations in mobile examination centers. In the seventh KNHANES, 31,689 individuals aged ≥1 year were selected, of which 24,269 individuals participated (participation rate: 76.6%). The KNHANES protocol was approved by the institutional review board of KCDC (2018-01-03-P-A), and prior consent was obtained from each participant. Oral examinations were performed by dentists from KCDC and public health dentists. Due to the limited number of public health dentists, oral examinations in the seventh KNHANES were performed on sub-groups. The health questionnaire survey data were used to assess the characteristics (e.g., patient sex, age, and subjective health) and unmet dental care needs of the cancer survivors, while the health survey and interview data were used to evaluate the oral health-related characteristics.

### 2.2. Exclusion Criteria

**Figure 1:**
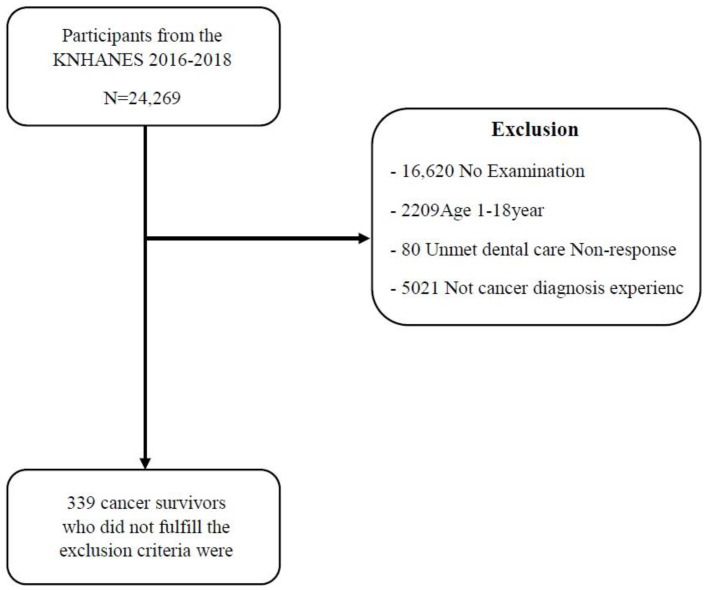
Participant flow diagram for final analysis.

A total of 24,269 people participated in the 2016–2018 KNHANES. Among them, 16,489 participated in the oral examination. Of the 16,489 patients, 2209 non-adult children and adolescents, 80 who did not respond to unmet dental care and 4941 who were not diagnosed with cancer by a doctor were excluded. Therefore, the final subjects of this study were 339 cancer survivors (Figure 1).

### 2.3. Cancer Survivors

The cancer survivor status was determined among adult participants of the seventh KNHANES, who had been diagnosed with cancer by a physician. Cancer survivors were classified based on the diagnosis of any one of the following cancers in the health survey: Gastric, liver, colorectal, breast, cervical, lung, thyroid, or other cancer.

### 2.4. Unmet Dental Care Needs

The participants were considered to have unmet dental care needs if their response to the following question was “Yes”: “In the past year, did you need any dental care (examination or treatment) that you were not able to receive?”

#### 2.4.1. General Characteristics

The participants’ sex (male and female), age (19–44, 45–64, and ≥65 years), education level (primary school or below, middle school, high school, and college or higher), monthly household income (upper, middle, and lower), subjective health (good, average, and poor), current smoker (yes and no), current drinker (yes and no), physical activities (yes and no), activity limitation (yes and no), and unmet healthcare needs (yes and no) were recorded. “Current smokers” were defined as those who smoked occasionally or daily. “Current drinkers” were defined as those who had consumed alcohol at least once in the past year. Exercise was defined as any aerobic physical activity.

#### 2.4.2. Oral Health-Related Characteristics

The oral health-related characteristics included the subjective oral health status, toothache, toothbrushing, frequency of toothbrushing, use of oral hygiene aids, chewing discomfort, and oral examination. Subjective oral health was determined based on the response to the question “How would you rate your oral health, such as your teeth and gums?” (Responses: Very good, good, average, poor, and very poor). Toothache was defined as a “Yes” response to the question “In the past year, did you experience any toothache?” Information regarding toothbrushing was obtained through the question “Did you brush your teeth yesterday?” The frequency of toothbrushing was classified as <3 or ≥3 times and as before breakfast, after breakfast, before lunch, after lunch, before dinner, after dinner, after snacking, and before going to bed. The use of oral hygiene aids was identified based on the use of products other than a toothbrush and toothpaste, such as dental floss, interdental toothbrush, and electric toothbrush. Chewing discomfort was identified based on the responses “much discomfort” or “some discomfort” to the question “Do you experience discomfort when chewing food due to a current problem with your teeth or dentures, gums, or mouth?” (Responses: Much discomfort, some discomfort, average, no discomfort, no discomfort at all). The patient was considered to have undergone an oral examination based on a “Yes” response to the question “In the past year, have you received an oral examination to check your oral health status even though you did not have any particular problem with your mouth?”

### 2.5. Statistical Analysis

The collected data were analyzed using SPSS 26.0 (IBM Corp., Armonk, NY, USA). All statistical analyses were performed considering the complex sampling method. The unmet dental care needs according to the characteristics of the participants were analyzed using descriptive statistics and Rao–Scott chi-squared test. The factors that were significant in the descriptive analysis and Rao–Scott chi-square test were analyzed by logistic regression to identify the factors that were not satisfied with dental care according to their characteristics. The factors associated with unmet dental care needs according to the characteristics were analyzed using logistic regression analysis. For all analyses, the statistical significance level was set to *p* < 0.05.

## 3. Results

### 3.1. Unmet Dental Care Needs According to the General Characteristics of Cancer Survivors

A total of 339 cancer survivors participated in this study. In this study, 29.5% of cancer survivors (men: 27.7% and women: 30.4%) had experienced unmet dental care needs. Concerning the monthly household income, 39.0%, 19.2%, and 22.7% of those in the lower, middle and upper groups, respectively, had experienced unmet dental care needs and the differences between these groups were statistically significant (*p* = 0.011). With respect to the subjective health status, 23.4%, 31.4%, and 30.1% of those who believed their health to be good, average, and poor, respectively, had experienced unmet dental care needs and the differences between these groups were statistically significant (*p* = 0.006). In addition, 48.3% of those with and 26.3% of those without activity limitations experienced unmet dental care needs (*p* = 0.003). Among the cancer survivors, 74.7% of those with unmet healthcare needs had experienced unmet dental care needs, while only 26.1% of those without any unmet healthcare needs had experienced unmet dental care needs, and the differences between these groups were statistically significant (*p* < 0.001). Meanwhile, there were no statistically significant differences in unmet dental care needs according to sex, education level, smoking status, drinking status, and physical activity status (Table 1).

### 3.2. Unmet Dental Care Needs According to the Oral Health-Related Characteristics of Cancer Survivors

With respect to the subjective oral health status, 19.2%, 20.4%, and 39.5% of those who believed their oral health to be good, average, and poor, respectively, had experienced unmet dental care needs, and the differences between these groups were statistically significant (*p* = 0.006). The experience of unmet dental care needs was found in 28.4% of those who brushed their teeth the day before and 73.1% of those who did not, and the differences between both groups were statistically significant (*p* = 0.017). Concerning the use of oral hygiene aids, the experience of unmet dental care needs was found in 32.5% of those who did not use dental floss and 34.2% of those who did not use mouth wash, and the differences between both groups were statistically significant. Moreover, 47.5% of those with and 21.9% of those without chewing discomfort had experienced unmet dental care needs (*p* < 0.001), while 14.2% of those did and 39.6% of those who did not receive an oral examination within the past one year had experienced unmet dental care needs (*p* < 0.001) (Table 2).

### 3.3. Factors Associated with Unmet Dental Care Needs among Cancer Survivors

Logistic regression analysis was performed to identify the factors associated with unmet dental care needs among cancer survivors using variables with a significance level of <0.05 in the simple analysis (Table 3). The results showed that the experience of unmet dental care needs was higher among those with activity limitation than among those without activity limitation (odds ratio [OR] = 2.14, 95% confidence interval [CI] = 1.04–4.40). Moreover, the experience of unmet dental care needs was lower among cancer survivors who did not have experience of unmet healthcare needs relative to those who did (OR = 0.17, 95%CI = 0.05–0.56). Furthermore, the experience of unmet dental care needs was much higher among those who did not receive an oral examination within the past year than in those who did (OR = 2.49, 95%CI = 1.22–5.07).

## 4. Discussion

This study investigated the experience of unmet dental care needs among Korean cancer survivors. The findings of the study revealed that 29.1% of Korean cancer survivors had experienced unmet dental care needs. According to previous studies, the rate of experiencing unmet dental care needs was 19.6% among older Korean adults [14] and 19.5% among Korean older adults living alone [15]. Since the findings showed a higher rate of experience of unmet dental care needs among cancer survivors than in other vulnerable populations, unmet dental care needs among cancer survivors can be considered an important oral health issue.

According to the study findings, cancer survivors with activity limitations were more likely to experience unmet dental care needs than those without activity limitations. Patients with activity limitations have lower accessibility to dental care services due to health issues or physical/mental impairment [16]. Moreover, cancer survivors experience physical adverse effects of emotional confusion due to cancer diagnosis, surgery, chemotherapy, and radiotherapy, with an overall decline in the quality of life [17] that may lead to activity limitation. Activity limitation can hinder participation in economic activities and affect income level [18], which further reduces the ability of the patients to bear medical care costs and limits their access to healthcare services. Therefore, cancer survivors are expected to face many limitations in using oral care services due to the aforementioned sequelae.

Cancer survivors who did not receive an oral examination within the past year were more likely to experience unmet dental care needs than those who did. While a direct comparison to the present study may be difficult, another study on the general population reported that a regular health check-up experience was an influencing factor for the quality of life. These results may reflect the psychological satisfaction that patients undergoing health check-ups experience due to benefits, such as early disease detection [19]. These findings suggested that people who receive an oral examination are more confident about their oral health and recognize the benefits of examination, and their unmet dental care needs can be resolved by visiting a dental clinic.

Cancer survivors who did not experience unmet healthcare needs were significantly less likely to experience unmet dental care needs than those who did. According to a study, 10.6% of Korean adults had experienced unmet healthcare needs, and the causes “because the symptoms were mild” (25.7%) and “economic reasons” (23.3%) accounted for almost half of them [20]. Oral diseases generally have relatively lower severity than other diseases, and as a result, oral diseases are less likely to impact the patient’s livelihood. Moreover, because a dental clinic is not a healthcare institution that patients visit for critical diseases that directly determine life or death, patients who have experienced unmet healthcare needs may not have recognized the graveness of dental diseases due to mild symptoms [14]. Furthermore, dental care services in Korea are often expensive and are not covered by insurance. The out-of-pocket cost can reach up to 83.5%. Therefore, dental care services represent very high cost and low coverage services among all health services [21]. Consequently, cancer survivors who experienced unmet healthcare needs may have also experienced unmet dental care needs due to economic reasons.

This study had some limitations. As a cross-sectional study, the temporality of how each factor influenced unmet dental care needs among cancer survivors could not be identified. In addition, because the experience of unmet dental care needs was self-reported, cancer survivors who needed dental care but did not recognize such need may have been excluded. As a result, there were limitations in the accuracy and objectivity of the measurements of unmet dental care needs. In addition, the data used in this study come from a questionnaire for the entire population of Korea, not a specific questionnaire for cancer survivors. Therefore, there are no data on confounding factors that are important for cancer survivors. Despite these limitations, this study is significant because it used a complex sample survey based on nationally representative KNHANES data to derive findings that can be generalized to all cancer survivors in Korea. Additional studies are needed to specify the causes of unmet dental care needs according to unmet healthcare needs among cancer survivors to identify the associated determinants.

## Figures and Tables

**Table 1:** Unmet dental care needs according to the general characteristics of cancer survivors.

Variable	Unmet Dental Care Needs		*p*-Value
	Yes	No	
Total	105 (29.5)	234 (70.5)	
Sex			0.684
Men	32 (27.7)	89 (72.3)	
Women	73 (30.4)	145 (69.6)	
Age (years)			0.076
19–44	7 (18.1)	30 (81.9)	
45–64	43 (26.1)	103 (73.9)	
≥65	55 (36.2)	101 (63.8)	
Education level			0.934
Primary school or below	45 (32.2)	77 (67.8)	
Middle school	12 (27.6)	26 (72.4)	
High school	26 (27.9)	66 (72.1)	
College or higher	22 (28.7)	65 (71.3)	
Monthly household income			0.011
Lower	66 (39.0)	103 (61.0)	
Middle	16 (19.2)	44 (80.8)	
Upper	23 (22.7)	87 (77.3)	
Subjective health			0.006
Good	14 (23.4)	51 (76.6)	
Average	44 (31.4)	107 (68.6)	
Poor	47 (30.1)	76 (69.9)	
Current smoker			0.152
No	94 (28.7)	221 (71.2)	
Yes	11 (42.6)	13 (57.4)	
Current drinker			0.231
No	70 (27.1)	156 (72.9)	
Yes	35 (34.0)	78 (66.0)	
Physical activity			0.803
No	65 (28.9)	136 (71.1)	
Yes	40 (30.4)	98 (69.6)	
Activity limitation			0.003
Yes	27 (48.3)	28 (51.7)	
No	78 (26.3)	206 (73.7)	
Unmet healthcare needs			0.000
No	90 (26.1)	227 (73.9)	
Yes	15 (74.7)	7 (253)	

Data are expressed as unweighted number (weighted %); tested using the Rao–Scott chi-square.

**Table 2:** Unmet dental care needs according to the oral health-related characteristics of cancer survivors.

Variable	Unmet Dental Care Needs		*p*-Value
	Yes	No	
Subjective Oral Health			0.006
Good	4 (19.2)	20 (80.8)	
Average	30 (20.4)	110 (79.6)	
Poor	71 (39.5)	104 (60.5)	
Toothache			0.882
No	53 (29.1)	140 (70.9)	
Yes	52 (30.0)	94 (70.0)	
Toothbrushing (day before)			0.017
No	7 (73.1)	2 (26.9)	
Yes	98 (28.4)	232 (71.6)	
Oral hygiene aid use (dental floss)			0.043
No	89 (32.5)	180 (67.5)	
Yes	16 (19.3)	54 (80.7)	
Oral hygiene aid use (interdental brush)			0.340
No	92 (30.5)	195 (69.5)	
Yes	13 (23.1)	39 (76.9)	
Oral hygiene aid use (mouth wash)			0.026
No	83 (34.2)	161 (65.8)	
Yes	22 (20.3)	73 (79.7)	
Oral hygiene aid use (electric toothbrush)			0.328
No	103 (30.0)	227 (70.0)	
Yes	2 (15.4)	7 (84.6)	
Oral hygiene aid use (others)			0.672
No	98 (29.3)	221 (70.7)	
Yes	7 (34.3)	13 (5.7)	
Chewing discomfort			0.000
No	45 (21.9)	181 (78.1)	
Yes	60 (47.5)	53 (52.5)	
Oral examination			0.000
No	82 (39.6)	121 (60.4)	
Yes	23 (14.2)	113 (85.8)	
Frequency of toothbrushing (3/day)			0.090
No	67 (34.5)	117 (65.5)	
Yes	38 (23.8)	117 (76.2)	

Data are expressed as unweighted number (weighted %); tested using the Rao–Scott chi-square.

**Table 3:** Factors associated with unmet dental care needs among cancer survivors.

Variables	Model1		Model2	
	OR	95%CI	AOR	95%CI
Sex				
Men	0.89	0.47–1.45	0.52	0.24–1.15
Women	1.00		1.00	
Age (years)				
19–44	0.39	0.15–1.03	0.92	0.28–3.04
45–64	0.622	0.35–1.11	0.73	0.36–1.48
≥65	1.000		1.000	
Monthly household income				
Lower	2.18	1.14–4.17	1.25	0.57–2.77
Middle	0.81	0.34–1.91	0.74	0.32–1.75
Upper	1.00		1.00	
Activity limitation				
Yes	2.62	1.39–4.94	2.14	1.04–4.40
No	1.00		1.00	
Unmet healthcare needs				
No	0.12	0.04–0.34	0.17	0.05–0.56
Yes	1.00		1.00	
Subjective oral health				
Poor	0.37	0.09–1.50	0.41	0.12–1.43
Average	0.39	0.22–0.70	0.52	0.25–1.07
Good	1.00		1.00	
Toothbrushing (day before)				
No	6.86	1.14–41.36	5.30	0.72–39.04
Yes	1.00		1.00	
Use of dental floss				
No	2.02	1.01–4.02	0.99	0.45–2.16
Yes	1.00		1.00	
Use of mouth wash				
No	2.04	1.08–3.86	1.57	0.80–3.11
Yes	1.00		1.00	
Chewing discomfort				
No	0.31	0.17–0.56	0.61	0.27–1.35
Yes	1.00		1.00	
Oral examination				
No	3.95	2.07–7.53	2.49	1.22–5.07
Yes	1.00		1.00	

OR, odds ratio; CI, confidence interval; AOR, adjusted odds ratio; tested using Model1 logistic regression, tested using Model2 multiple logistic regression.

## Data Availability

Data are available on the official KNHANES website (https://knhanes.kdca.go.kr/knhanes/sub03/sub03_02_05.do, accessed on 1 June 2022).
